# Economic Inequities in the Application of Neuromodulation Devices

**DOI:** 10.7759/cureus.5685

**Published:** 2019-09-17

**Authors:** James Leiphart, Megan Barrett, Mahesh B Shenai

**Affiliations:** 1 Neurosurgery, Inova Neuroscience Institute, Falls Church, USA

**Keywords:** neuromodulation, household income, spinal cord stimulation, economic inequity, vagal nerve stimulation, medical economics

## Abstract

Background

There is a significant upfront cost for the use of neuromodulation devices. The high cost of these devices may lead to disproportionate application in geographical regions with different levels of financial resources. The purpose of this study was to determine if there is geographic based economic inequity in the application of neuromodulation devices in the United States.

Methods

Population and average household income data by county from the year 2010 were obtained from publicly available databases on the US Census website. The number of stimulators sold by county in the years 2009 and 2010 were provided by two of the four neuromodulation companies with commercially available products. Pearson correlation and t-test statistics were performed.

Results

Of the 3142 U.S. counties analyzed, only 689 placed neuromodulation devices during this period of time. There was a difference in average household income between counties with device implants ($49,663) and counties with no device implants ($41,314), which was statistically significant (p<0.001).

Conclusion

Analysis of neuromodulation devices placed in 2009 and 2010 from 50% of neuromodulation companies demonstrated that there was an income disparity between counties in which implantation of devices occurred and counties in which there were no device implantations.

## Introduction

 Neuromodulation devices are used for the treatment of a variety of neurological disorders. These devices have been approved by the United States Food and Drug Administration for the treatment of one or more indications, and each device has data from one or more randomized prospective clinical trials substantiating its efficacy. Neuromodulation devices include the vagus nerve stimulator for epilepsy and depression, the deep brain stimulation for Parkinson’s disease, essential tremor, dystonia and obsessive-compulsive disorder, the spinal cord stimulation for chronic intractable pain, and the intrathecal pump for chronic intractable pain and spasticity [1-11]. The long-term benefits of many of these therapies in reducing overall costs of care have been demonstrated [12-16]. All of these devices have significant upfront costs that may present a barrier to their use. Any time that the cost of therapy is high, there is a concern that inequities in its utilization will occur based on socioeconomic factors.

Lower socioeconomic status has been correlated with lower utilization of general medical care, non-neuromodulation implanted devices, and implanted neuromodulation devices [17-30]. United States government health care policy has been developed with the intention of delivering equal and comprehensive care to the entire population regardless of socioeconomic or other factors [19]. However, cost control measures have necessarily led to expenditure limitations, and these limitations have the potential to create a disparity in access between patients with private insurance and patients who rely on government programs to pay for their health care or who are completely uninsured. This study was designed to evaluate variability in the utilization of neuromodulation devices based on socioeconomic factors. 

## Materials and methods

Data on stimulator implantations were obtained from two of the four companies producing FDA approved neuromodulation devices at the time of the study. Company data were used rather than data available on public websites, as it would represent a more comprehensive population, not limited to Medicare or Medicaid payors. The data obtained from the companies include all implanted patients in the period of analysis regardless of the method of payment for care or inability to pay for care. Zipcodes from patients' living addresses were used as the county location correlating with household income data, to reflect patients' socioeconomic status, as opposed to the location of the implanting hospital. All four neuromodulation device companies were contacted, Boston Scientific, Cyberonics, Medtronic Inc., and St. Jude Medical, with a request for de-identified data grouped by county level. Two of the four companies provided data, while the other two companies did not respond to our request. As such, the data reflect the use of spinal cord stimulators and vagal nerve stimulators, but not deep brain stimulation. The participating companies provided the number of stimulators sold in the United States in the years 2009 and 2010 organized by county.

Population and average household income data are publicly available on the U.S. Census website. We obtained these data from the website organized by county for the year 2010, the most recent U.S. Census. Pearson correlation and t-test statistics were performed.

## Results

**Figure 1:**
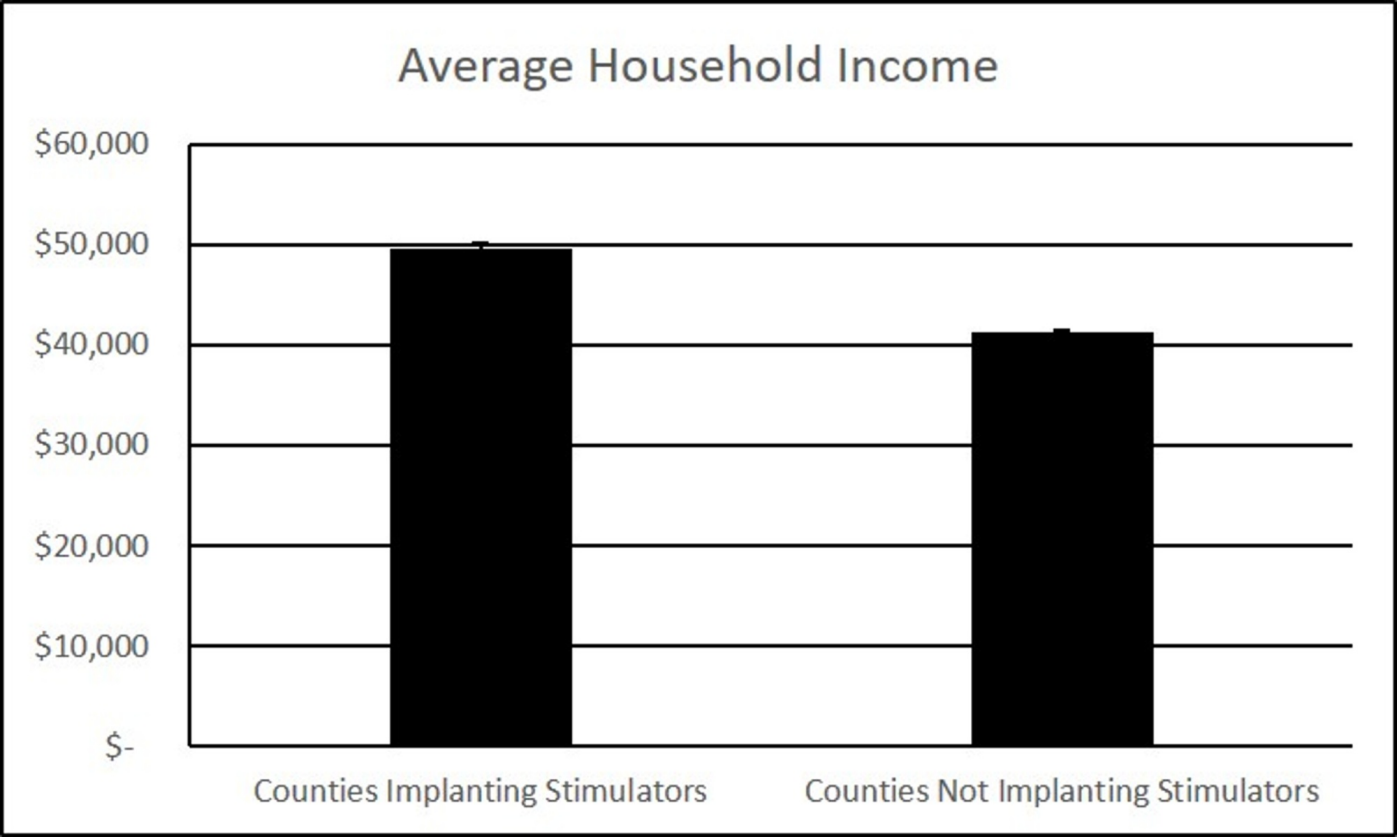
Average household income Comparison of average household income in counties implanting stimulators, versus counties not implanting stimulators

The data analysis included 3142 U.S. counties. Of these counties, 689 had placement of neuromodulation devices during the years 2009 and 2010. There was a statistically significant difference in the average household income, between implanted patients' counties and those counties without device implants (Figure 1, p<0.001). The average household income of the counties in which neuromodulation devices were implanted was $49,663. The average household income of counties that did not implant neuromodulation devices was $41,314. 

**Figure 2:**
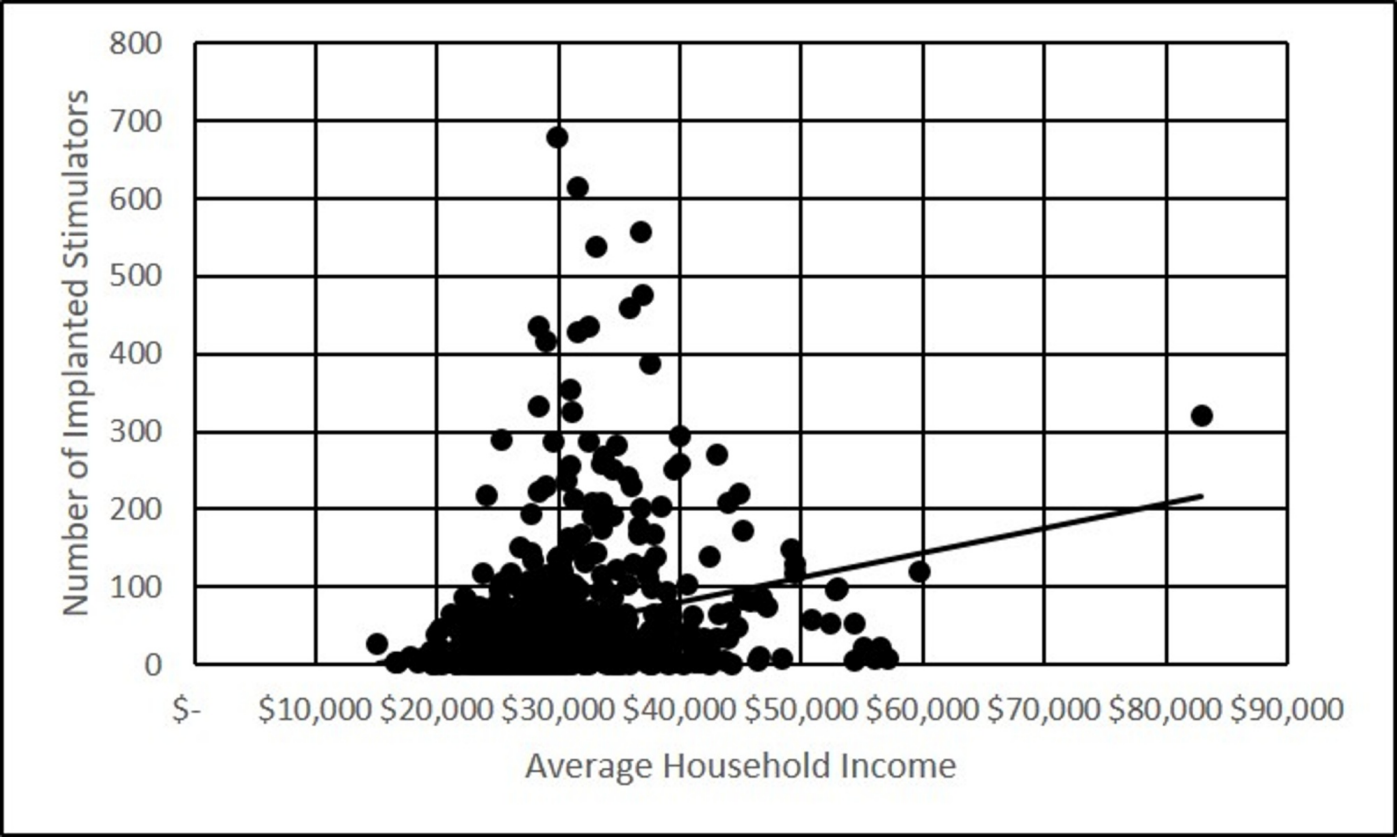
Number of implanted stimulators per county, versus the average household income Scatter plot depicting average household income and the number of stimulators implanted in 2009-2010, for each of the 689 counties included in the study

**Figure 3:**
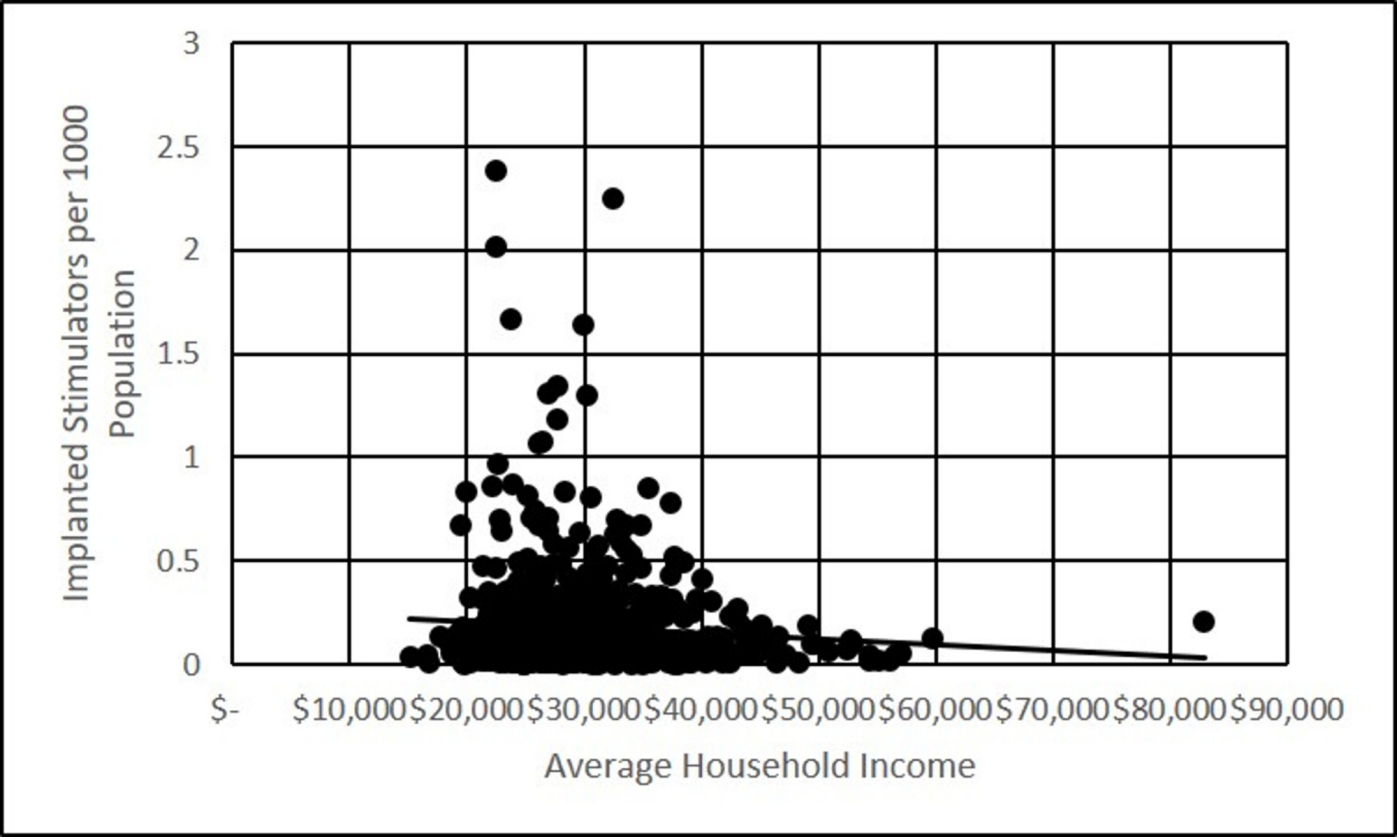
Implanted stimulators per 1000 people, versus average household income Scatter plot depicting the incidence (per 1000) of neurostimulator implantation in 2009-2010, versus average household income for each of the 689 counties included in the study

Pearson correlations were performed on the data from the 689 counties that implanted neuromodulation devices in the years 2009 and 2010. There was no correlation between average household income and number of devices placed (Figure 2, p>0.01). There was a negative correlation between average household income and devices placed per 1000 population (Figure 3, p<0.001). 

## Discussion

This analysis demonstrates a higher average household income in counties with neuromodulation device implantations than in counties without implantations. This finding would seem to suggest a disparity of access due to socioeconomic factors, however, there are covariates of higher average household income that could be responsible for this effect. Higher density urban areas tend to have higher household incomes commensurate with the higher cost of living. Additionally, counties with higher populations are also more likely to have higher acuity hospitals with subspecialty presence. With these presumptive correlations of population density with income and access, any of these factors could be responsible for the income disparity found between implanting and non-implanting counties. 

Based on the data, there was no statistically significant correlation between the average household income and the number of devices implanted by county, however, there was statistically significant negative correlation when implantations were normalized to the population (per 1000). This finding would imply that high-income populations are actually less likely to require device implantations. However, this conclusion is itself confounded by the differential prevalence of various neuromodulation indications in income segments. 

The correlation between average household income and per capita device implantations in the counties with implantations was negative. This could argue against a negative effect of lower socioeconomic status on access to neuromodulation devices, but the finding is difficult to explain. The highest population counties in the United States, Cook County, Illinois for example, have some of the highest disparities in income. Therefore, the higher average household income statistic in the county is driven by a small population of very high-income households, but there is also a large population of low-income households. If the negative per capita implantation correlation is driven by the larger population of low socioeconomic status within those counties, the data would not appropriately demonstrate a disparity. 

Determining if there is any subtle effect of socioeconomic factors on the utilization of medical technologies is especially important in the current environment of federal health care policies moving toward universal coverage. One method to achieve universal coverage is the expansion of Medicare to cover the uninsured. Medicare plans may not cover the entire cost of a high priced device like a neuromodulation device. If the payer does not cover the entire cost of the device, hospitals providing the surgical implantation may decide not to provide these procedures. The findings of this study could be interpreted to indicate that this is occurring now, and that medical systems in counties more reliant on Medicare services are not making these devices available. This should encourage government payers to cover the costs commensurate with the implantation of neuromodulation devices and provide equity in the access to their benefits.

But what is the evidence for this? Is the disparity in access to neuromodulation devices by average county income due to the difference in private insurance, public insurance and no insurance, or is it an effect of lower income itself? One study demonstrated that health insurance status had more influence than income on disparities in patient care [19]. A disparity in the resources of mammogram facilities was found between uninsured/publicly insured patients and privately insured patients [18]. Lack of health insurance is associated with less screening for breast and cervical cancer, and Medicaid and uninsured patients have higher mortality following major surgery than privately insured patients [20-21]. Medicaid patients are less likely than privately insured patients to get parenteral analgesics and sedatives in emergency departments [22]. Medicaid and self-insured patients receive fewer pacemakers, and Medicaid use, not low income, is associated with fewer deep brain stimulator implantations [24,28]. These studies suggest that public insurance may be a barrier preventing patient access to these technologies.

On the other hand, some studies have found income and other factors to be responsible for disparities in care rather than insurance status. Patients with low financial status had fewer reports of good service or collaborative care than patients with higher financial status [23]. Economic status is frequently tied to geography, so many studies have found the two factors intertwined in access to care. Patients in lower-income zipcodes are less likely to have a referral for cochlear implant [26]. Disparities in access to stroke care fall along geographic/economic and urban/rural lines, with low income and rural areas having less access [17]. Studies have shown that both spinal cord stimulator surgery and deep brain stimulator surgery are more common in urban than rural areas [29-30]. There is even a geographic/economic disparity in deep brain stimulation surgery use among patients with Medicare insurance where patients in higher socioeconomic zip codes are more likely to get deep brain stimulation therapy [27]. A study in the Canadian health care system, which is a publicly funded medical system, found a higher cardioverter-defibrillator implantation rate in patients living in higher socioeconomic neighborhoods [25]. This is not due to inequity in payment for these devices but rather represents more complex socioeconomic factors underlying the disparity. 

## Conclusions

This study demonstrates geographic variability in the utilization of neuromodulation devices. There is some suggestion that this variability may fall along socioeconomic lines with less utilization in lower socioeconomic areas. Other studies have demonstrated similar disparities in medical care and have attributed them to insurance status, income, or both. The preponderance of evidence in this direction indicates that more research is needed to determine the underlying causes and greater efforts are needed to assure equity in patient care.
